# Bishop Coxe on Science

**Published:** 1869-04

**Authors:** 


### Miscellaneous.

### Bishop Coxe on Science.

We have seen in several prominent newspapers, including one of the respectable journals of this city, extracts from a report in the *Buffalo Christian Advocate* of a lecture by Bishop Coxe of Buffalo. The lecture was on the "Connection of Science and Revealed Religion;" and we are told that it was "full of astonishing facts." It most assuredly was, if the report is an accurate one. For instance, we are informed in it "that the crust of the earth is *just* twenty-one miles thick; that the world's conflagration has already begun, as *the internal fires are tending rapidly to its annihilation;* that earthquakes have multiplied since the Christian era, and *indicate a speedy collapse of our system;* that if the Lisbon earthquake had been a *little* more severe the shock would have *driven out* that portion of the surface; and, *most wonderful of all*, that on the first day of the present century, a little planet was discovered by Kepler in our system; and since then a large family of these little planets have appeared, which are *parts of an exploded world*, and that some of these fragments have fallen on the earth in *showers of meteors.*" After stating this last wonderful fact, the lecturer appears to have made the sapient inquiry, "If Kepler's planet exploded, *why may not ours?*"

It is pleasant to know that scientific men, who have hitherto estimated the thickness of the earth's crust all the way along from ten miles up to eight hundred, are now agreed that it is *just* twenty-one miles. It is well to have these disputed questions finally settled. It is not so pleasant, however, to be informed that the internal fires of our planet, instead of cooling down by slow degrees, as many of the most eminent students of physics have assumed, are raging more and more fiercely, and that "a speedy collapse" of the globe is to be expected. It is also unwelcome news that the strain upon the earth's crust by the Lisbon earthquake has been so exactly measured, and that a little more force would have "driven out" a piece of it; for we cannot tell how fearful may be the results of the next great earthquake, which, from the increase of earthquakes, may be expected to be "a little more severe." The consequences *may* be such an explosion of our terrestrial ball as the bishop darkly hints at in an interrogatory way.

But concerning the last of these facts, and the "most wonderful of all," we must venture to express a doubt. There is pretty good reason to believe that Kepler died in the year 1630; and we must still adhere to the commonly accepted statement that it was another man who discovered the little planet Ceres on the first of January, 1801. It is true that Olbers advanced the theory that this little planet, and the others like it which were soon afterwards discovered, are parts of a shattered planet; but we supposed that this theory was now almost universally rejected by astronomers. These planets now number about a hundred, and are scattered through a belt of the heavens more than a hundred millions of miles in breadth, or more than the distance from the earth to the sun; and it is difficult to conceive of an explosion that could throw great fragments of a huge planet through distances so vast. There are other objections to the theory which are even more serious. On the other hand, if we accept the nebular hypothesis (of which we gave a sketch in the last *Journal*,) the formation of this zone of minor planets is as readily explained as the origin of our own globe, and the mightier orbs that move in wider circles round their solar center.

Of course we do not believe that the lecturer said that Kepler discovered Ceres. He probably mentioned Kepler in that connection, and the reporter, as reporters sometimes do, got things somewhat mixed in his memoranda. But the good bishop very likely did make most of the other statements ascribed to him; for it is a common vice of unscientific writers on scientific subjects to be very rash and dogmatic on doubtful or disputed points. They frequently have no hesitation in deciding questions which wise men, who have made them the study of their lives, do not venture to consider settled.

Even if the lecturer made none of the statements in the positive form in which they are reported, there can be no doubt that the *Christian Advocate*, and the papers that have copied its report of the lecture, are responsible for the promulgation of these errors and absurdities; and it is certainly to be regretted that there is so general an ignorance of the very alphabet of physical science that these absurd things can be printed, and reprinted, and widely read without challenge or contradiction.—*Boston Journal of Chemistry.*

